# Two Cases of Poisoning and Two Cases of Surgery

**Published:** 1866-09

**Authors:** Hiram Wanzer

**Affiliations:** Chicago


*Two Cases of Poisoning and Two Cases of Surgery.* Related to the Chicago Medical Society, Aug. 3d, 1866, by HIRAM WANZER, M. D., Chicago.

CASE I. POISONING WITH LAUDANUM.

A. C., aged 25, at midnight, May 3d, 1866, swallowed two ounces of laudanum. Soon after the occurrence, was taken to his home. Nearly an hour elapsed before I saw him. I found him in a state of profound insensibility; the skin covered with cold sweat; the pulse slow and moderately full; the pupil of the eye contracted; the breathing stertorous. There was preternatural heat of the head, indicating cerebral congestion. I applied cold water immediately to the scalp. After the application had been continued for half an hour, reaction and consciousness was sufficiently established for the administration of an emetic. The stomach was so paralyzed, that it was not until large quantities of warm water had been drank, assisted by mechanical irritation of the fauces, that emesis was induced. After the act of vomiting, he relapsed again into his coma. There had been sufficient of the poison absorbed into the blood to prolong the narcotism for twelve hours. He was aroused every hour to take five grains of carbonate of ammonia in emulsion. This, I believe, exerted a powerful influence in establishing a more complete reaction. Thirty hours after taking the drug, his health being quite re-established, he left the city.

### CASE II. POISONING WITH CORROSIVE SUBLIMATE.

On the 30th of May last, I was called to see Mrs. W——, aged 35. She had swallowed two ounces of a solution of corrosive sublimate, which contained three grains of the mineral. The prompt action of an emetic, and the administration of the white of eggs and other albuminous substances, rendered the remaining poison so inert, that she suffered no material harm from her imprudence.

### CASE III. ANKYLOSIS OF THE KNEE-JOINT.

A Swede, master of a vessel, aged 25, called at my office, 27th of April last, with extra articular ankylosis of the left knee-joint. He stated that in the month of January last he had been struck with an axe, which caused a penetrating wound one inch and a half in length, at the inner edge of the patella, resulting in the immediate escape of the synovial fluid. Active synovitis and complete immobility of the joint followed. The limb was placed in permanent extension. The want of flexion, resiliency and mobility of the joint seemed to have depended upon plastic deposit and chronic thickening in the synovial membrane and fibrous textures of the joint. Even the quadriceps tendon and hamstring muscles had participated in the inflammatory action and plastic effusion, they being in a state of great rigidity and soreness. The superficial structures of the joint were tender and œdematous. There was an enduring cicatrix which somewhat embarrassed the future treatment, but will never impair the usefulness of the joint. All medical treatment having failed, and fearing the case would, at no distant period, result in true permanent osseous ankylosis—upon his first visit, I seized the limb and made forcible flexion. I succeeded at this time in breaking up most of the adhesions. After the operation, daily passive motion was enjoined. Frictions were made with chloroform liniment, alternated with the hot salt douche. We have succeeded in restoring perfect mobility to the limb. The inflammatory deposits appear to have been absorbed and the joint restored to its normal size and functions. The man now walks with ease; is pursuing his accustomed vocation.

### CASE IV. PENETRATING WOUND OF THE ABDOMEN AND INTESTINES—RECOVERY.

A. B., a boy, 9 years old, of English birth, July 26th last, had ascended a cottonwood tree to the height of twenty-five feet. A broken branch, upon which he was standing, gave way, precipitating him upon a picket fence situated under the tree. Three of the pickets inflicted severe injuries upon the body. The first, striking the upper third of the left tibia, made an oblique indentation of the bone upon its cancellated tissue, two inches in length, without fracture. There was also a severe contusion over the crest of the right tibia, at the junction of the middle with its upper third. The second picket entered the left axillary space, making an extensive wound without entering the thoracic cavity. The third picket entered the left inguinal region, dividing the structures transversely across this region and the hypogastric, peeling up the tissues from the muscles about three inches in a valvular manner. The wound through the abdominal muscles was circular, and did not exceed one inch and a half in diameter. The peritoneum and one of the coils of the intestines were lacerated. The rent in one of the convolutions of the ilium was an inch; the aperture ragged, and the mucous membrane everted. There was a small spot upon the ilium, near the injured part, that looked dark and gangrenous, and which I was apprehensive might sphacelate.

I saw the patient twenty minutes after the accident. The protruding mass lying outside was the size of a large orange. There was also hernia between the abdominal muscles. There seemed to be a sort of artificial pouch in the cellular substance between the fascia transversalis and the transversalis muscle, containing as much intestine as protruded externally. Pulsation of one of the mesenteric arteries was visible, but there was little hemorrhage.

Dr. W. R. Marsh having given chloroform to anæsthesia, the legs being flexed, and the abdominal muscles relaxed as much as possible, with tepid water I gently washed the fecal matter and other foreign substance from the wounded structures, coapting the edges of the wound of the intestine with continuous sutures, cutting the same off closely. The protruding mass was then returned by taxis. I found that reduction through the small aperture in the abdominal muscles by taxis, in the axis of the tumor, seemed impossible without enlarging the opening; but by steadying the tumor outside with the left hand, I introduced the index and middle fingers of the right within the constriction, and by gentle pressure, little by little, returned the hernial protrusion with as cautious manipulation as possible. Ascertaining by the touch within that the viscera were all in their proper position, the external wound was closed with several interrupted sutures, a compress and bandage. A single suture was all that was necessary for the wound in the axilla.

The patient suffered during the first twenty-four hours from shock. In forty-eight hours after, there was preternatural heat and some tenderness and fulness of the abdomen, indicating the approach of fatal peritonitis. In a few hours those unhappy symptoms all disappeared. The wounds nearly healed by the first intention. The patient complained exceedingly at times during the first seven days, of vesical tenesmus, when the bladder became in the least distended. During this time, there was also much thirst and febrile reaction. The treatment during the first ten days, was one quarter of a grain of opium every four hours. The sutures were allowed to remain undisturbed. Cold water was applied continuously to the abdomen. The diet was exclusively liquid, such as could be absorbed into the blood with as little fecal residue as possible, such as milk, barley water, chicken broth, and beef essence.

The bowels were not moved for fifteen days, at the end of which time he complained of pain. An oleaginous enema was then given which resulted in a large passage of impacted feces. Twenty days have now elapsed, and we regard the patient out of danger.

